# Dengue Vaccines Regulatory Pathways: A Report on Two Meetings with Regulators of Developing Countries

**DOI:** 10.1371/journal.pmed.1000418

**Published:** 2011-02-22

**Authors:** Richard Mahoney, Liliana Chocarro, James Southern, Donald P. Francis, John Vose, Harold Margolis

**Affiliations:** 1 Dengue Vaccine Initiative, International Vaccine Institute, Seoul, Korea; 2 World Health Organization, Department of Immunization, Vaccines and Biologicals, Regulatory Pathways, Secretariat to the Developing Country Vaccine Regulators' Network (DCVRN), Geneva, Switzerland; 3 DCVRN, Geneva, Switzerland; 4 Global Solutions for Infectious Diseases, Brisbane, California, United States of America; 5 Dengue Branch, Division of Vector Borne Infectious Diseases, United States Centers for Disease Prevention and Control, Puerto Rico, United States of America

## Abstract

Richard Mahoney and colleagues summarize two recent meetings convened by the Pediatric Dengue Vaccine Initiative and the Developing Countries' Vaccine Regulators Network on regulatory issues that need to be addressed before licensing dengue vaccines.

**Summary Points**

- Because a dengue vaccine should be tetravalent in nature and provide protection against all four dengue serotypes, regulatory agencies need to address additional issues associated with multi-valent vaccines such as interference between the vaccine serotypes.
- Safety assessment needs to account for the potential risk of inducing antibody-enhanced diseases (antibody-dependent enhancement).
- Because of the varying epidemiology and disease impact in different countries and regions, dengue vaccines will likely need to be evaluated in diverse populations initially in both the Americas and the Asia Pacific region.
- Several national regulatory authorities (NRAs) in endemic developing countries are likely to be engaged in review of both applications for clinical evaluation and for marketing of vaccines and they should receive support as appropriate.
- Manufacturers can submit a dossier to the European Medicines Agency for the Evaluation of Medicinal Products (EMA) for review (Scientific Opinion). This is possible due to the introduction of Article 58 of EMA's regulation 726/2004 (within which the example of dengue is specifically mentioned). This Opinion could facilitate the review process by NRAs in developing countries. Manufacturers may also obtain scientific advice and protocol assistance from the EMA, which may facilitate later Article 58 review.
- The Developing Countries' Vaccine Regulators Network recommends that consideration be given to agreements for joint reviews of clinical trial applications by similarly affected NRAs and also the review of applications for licensure in order to accelerate the launch and introduction of dengue vaccines. The NRAs would need to have access to the necessary expertise to review the quality and safety aspects of the license application.
- It is critical that improved standardized tests be introduced as soon as possible for the diagnosis of early infection and for the measurement of immune protection (requiring identification of a correlate of protection). The World Health Organization (WHO), through its Expert Committee on Biological Standardization, can evaluate and standardize such tests; in addition, WHO and its Collaborating Centers may also help ensure availability of necessary standards and reagents for use in the field.

## Background

The Pediatric Dengue Vaccine Initiative (PDVI) is a product development partnership (PDP) based at the International Vaccine Institute (IVI) in Seoul, Korea, and is supported by the Bill & Melinda Gates Foundation. PDPs are nonprofit entities that seek to accelerate the development, evaluation, and introduction of vaccines, drugs, devices, diagnostics, and other technologies to reduce the burden of disease in developing countries. They operate through partnerships with public and private organizations to assemble networks with the needed skills for the work required to achieve the goal of disease reduction. PDPs have been formed for vaccines against malaria, HIV, tuberculosis, meningitis, and other diseases, including dengue. PDVI's mission is to reduce the burden of dengue disease by accelerating the development, evaluation, and introduction of safe, effective, and affordable dengue vaccines. The PDVI collaborated with the Developing Countries' Vaccine Regulators Network (DCVRN) to convene two meetings in 2007 concerned with the regulatory issues that will need to be addressed to license dengue vaccine(s). (As of January 1, 2011, the Pediatric Dengue Vaccine Initiative is renamed the Dengue Vaccine Initiative Consortium and is composed of IVI, the Initiative for Vaccine Research of the World Health Organization, the International Vaccine Access Center of Johns Hopkins University, and the Sabin Vaccine Institute.)

DCVRN is a World Health Organization (WHO) initiative involving nine countries: Brazil, China, Cuba, the Republic of South Korea, India, Indonesia, the Russian Federation, South Africa, and Thailand. It provides a forum for discussion, advancement of knowledge, and exposure to policies and procedures pertaining to oversight of clinical trials and evaluation of clinical data for registration of vaccines. The United States Food and Drug Administration (FDA) and the European Union European Medicines Agency for the Evaluation of Medicinal Products (EMA) often participate in meetings of the DCVRN. In addition, regulatory staff from several additional dengue-endemic non-DCVRN member countries (Cambodia, Malaysia, Philippines, and Vietnam) have participated in the dengue vaccine sessions at the DCVRN meetings.

The PDVI has also worked with the Initiative for Vaccine Research (IVR) of WHO to update guidelines for the clinical evaluation of dengue vaccines [1]. Because the work with IVR has been reported elsewhere [2], the rest of this paper will address only the work with DCVRN.

The first meeting with DCVRN was held in April 2007 in Brasilia, Brazil. The agenda included detailed information on the epidemiology of dengue, the nature of the disease, the status of development of dengue vaccines, and some of the regulatory issues that will need to be addressed. No vaccine developers participated in this meeting. The second meeting, held in Bangkok, Thailand, in December 2007, included several companies that are developing dengue vaccines. Each of these companies presented the development status of their candidates and outlined the issues that they felt were most important for testing and ultimate regulatory approval of these vaccines.

## Dengue Vaccine Development: The Regulatory Challenges

Thanks to substantial increases in funding from public and private sources, the pace of development of vaccines of concern to developing countries has recently accelerated. As the development of these vaccines proceeds, a number of regulatory issues arise.

Dengue is the world's most important arboviral disease, affecting over 3.6 billion people in 124 countries. It results in about 34 million cases of clinical dengue fever with more than 6% of cases developing more serious forms such as dengue hemorrhagic fever (DHF) and dengue shock syndrome (DSS), both of which can result in death [3]. There are no effective drugs to treat dengue infection [4]. Vector control for the mosquito vector, *Aedes aegypti*, has not proven to be sustainable or highly effective [5]. Dengue presents with fever, retro-orbital pain, myalgia, and petechia. Symptoms may last for 10–14 days and may progress to DHF and DSS, leading to death. While there are no therapeutic drugs, modern intensive care can reduce case fatality rates to less than 1%. However, there are no widely available or reliable rapid laboratory diagnostics for the first 4–5 days of infection, thereby limiting the ability of physicians to identify at-risk patients early in the disease [6]. There is consensus that use of an effective vaccine is the best option to control this disease [7].

Dengue is prevalent only in tropical countries where the vector is common. Clinical trials to assess the safety and efficacy of dengue vaccines must therefore be carried out in these developing countries, and it is likely that the first licensure of dengue vaccines will occur there.

Dengue diseases are caused by four related viruses (DENV 1, DENV 2, DENV 3, and DENV 4). In most countries, one virus tends to dominate during a season, but is replaced by other viruses over several years. Thus, a dengue vaccine must be effective against all four viruses, i.e., tetravalent. In addition, there is the theoretical possibility of an adverse immune response in individuals not protected against all four viruses. An individual protected against one or two of the four viruses may be subject to a severe immune response (antibody-dependent enhancement) if exposed to a virus against which the individual is not protected, although the only human studies to assess this possibility have not observed these events [8],[9]. The challenge of tetravalent vaccine development is compounded because of interference among the viruses and by the lack of an animal model for dengue infection and disease [6].

Dengue affects a wide age range from infants through young adults [10]–[12]. A dengue vaccine will have to be evaluated in diverse populations in both the Americas and the Asia Pacific region and will have to be delivered in national immunization programs to infants and in “catch-up” programs in older age groups.

In recognition of these several complications, there is high priority to ensure that developing countries have the capability to undertake appropriate regulatory review of proposed clinical studies and of applications for licensure. It is important that the national regulatory authorities (NRAs) of these developing countries have the information, training, and capabilities to review and approve applications to undertake clinical trials and eventually review and, if appropriate, approve applications for licensure of dengue vaccines. Involvement of the US FDA and the EMA can be helpful in assuring a high level of regulatory review. In particular, the EMA may be able to provide scientific advice and protocol design assistance and assist regulatory review through its Article 58 of regulation 726/2004.

## Status of Development of Dengue Vaccines

Dengue vaccines have been under development since the 1940s, but due to the limited appreciation of global dengue disease burden and of the potential markets for dengue vaccines, industry's interest languished throughout the 20th century [13]. However, in recent years the development of dengue vaccines has accelerated dramatically. This changed landscape is illustrated by the progress in clinical development of sanofi pasteur's live-attenuated tetravalent chimeric vaccine, which could be licensed as early as 2014.

It is fortunate that there are several vaccines under clinical development, because there is no certainty as to which, if any, of the candidate vaccines will prove to be safe and efficacious, and wide access will depend upon a competitive environment in which several manufactures are supplying vaccine. For a recent review of dengue vaccine development, see Whitehead et al. [14].

Vaccines in clinical development include four live attenuated vaccines and one subunit vaccine under development by Hawaii Biotech. (In July 2010, Merck Vaccines & Co. acquired the rights to the Hawaii Biotech vaccine and has stated its intention to continue development of the candidate.) One of the live attenuated vaccines (GlaxoSmithKline) is a traditional vaccine prepared by cell passage: three of the other live attenuated vaccines are genetic constructs and involve both the use of chimeras and of gene deletions. The vaccines under development by GlaxoSmithKline and sanofi pasteur (live attenuated yellow fever–dengue chimera) have completed Phase 1 testing and initial Phase 2 (Phase 2a) testing. The vaccine developed by the US National Institutes of Health (NIH) (dengue-dengue chimera with gene deletion) has been in Phase 1 testing under the auspices of NIH and entered tetravalent Phase 1 trials in 2010 under the auspices of NIH licensees: Biological E, Panacea, and Butantan. The sanofi pasteur vaccine entered expanded Phase 2 (Phase 2b) testing in 2009 in Thailand. The Hawaii Biotech vaccine entered Phase 1 testing of its monovalent DEN1 vaccine in 2009. A vaccine (dengue-dengue chimera) under development by Inviragen entered Phase 1 testing in 2010. If the Phase 2b clinical evaluation of the sanofi pasteur vaccine indicates that the vaccine is safe and effective, it is possible that licensure may occur as early as 2014. Partly because of the requirement for efficacy against four viruses, the exact specification and design of Phase 3 trials is unknown. There remains the possibility that a vaccine may be licensed on the basis of safety and efficacy data against one or perhaps two serotypes. The design of subsequent Phase 4 trials that may address efficacy (and safety) with respect to the other viruses is also unknown. For further discussion, see the WHO Guidelines [1],[2].

## Results of the PDVI/DCVRN Meetings on Dengue Vaccines

Selection of suitable sites for clinical trials of dengue vaccine candidates is important, and PDVI has helped to identify more than ten sites in developing countries. PDVI is working with these sites to enhance their capabilities in surveillance and to undertake clinical studies. Important results have been obtained from these sites ([15]–[17] and O. Wichmann, I. Yoon, S. Vong, K. Limkittikul, R. Gibbons, et al., unpublished data), and one site was chosen by sanofi pasteur for its Phase 2b trial in Thailand. Key criteria for consideration in selecting sites are investigator experience, dengue serotype prevalence, NRA competence, the implications of rural versus urban sites, availability of multi-year longitudinal data on dengue incidence, and the laboratory's ability to detect clinical dengue cases.

Safety monitoring of dengue vaccine trials will need to be especially diligent in the initial stages of trials because of possible immune enhancement in individuals a) who are only partially immunized and become naturally infected or b) who have been previously infected and receive a first vaccine dose. Improved definitions of adverse events following immunization are needed. For example, there is a need to define adverse events 1) caused by infection occurring between doses of vaccine, and 2) adverse events caused by natural virus infection. In addition, there is a need to validate methods to detect these events. Improved safety surveillance and early viral analysis in cases of fever are needed. These issues have been addressed for the Phase 2b trial in Thailand.

The NRAs in dengue-prevalent countries will be requested to consider new approaches to vaccine development that may accelerate the process, and the NRAs must ensure a favorable risk–benefit for the country. In Brasilia and Bangkok, there was a discussion on licensure of a tetravalent dengue vaccine with demonstrated efficacy and safety against only one serotype. One potential strategy, under these conditions, would be to require post-marketing surveillance for safety and efficacy against all four viruses.

It is critical that any vaccine being tested in subsequent studies be the same in all respects as that being used in earlier clinical stages. Any changes to the manufacturing or formulation steps could result in the need to repeat the clinical trials or at the least require complex bridging studies.

A common understanding of “standard definitions” is important, especially for lab-based serological tests. Standardized test methods and the acceptance of validated international reference standards for antibody responses and virus typing is essential. In addition, clearer definitions are needed for Phase 2a, Phase 2b, and Phase 3 trials.

Clinical trials should generate data to help a) understand the science of the possible severe immune enhanced disease and b) identify correlates of immunity/protection that would assist vaccine development. The development of an animal model would be of great benefit but likely not essential for licensure.

Further effort is required to define desirable characteristics of vaccine trial design. This is largely the responsibility of the manufacturers (in consultation with competent regulatory agencies) involved in vaccine development. These include target age group for immunization, vaccine dosage schedule, trial duration and follow-up, assessment of possible confounding affects of immunity to other flaviviruses, diagnosis and case definition, and long-term surveillance. Additional issues for assessing potential trial sites include the prevalence of the viral strains, influence of concurrent mosquito control programs, community involvement, and virological and diagnostic services.

Phase 3 trials may be undertaken in some countries based on the safety and efficacy data from Phase 2 (a or b) trials in other countries. Responsible NRAs need to establish procedures for review and acceptance of second-country data and will need to assess formulation or regimen changes dictated by prior clinical trials.

The DCVRN favors the development of formal procedures for collaboration and joint review of clinical trial applications and monitoring including good clinical practices reviews and inspections by the responsible NRAs, EMA, and/or US FDA with facilitation by WHO. The cooperation of the sponsor will be required. The NRAs would need to have access to the necessary expertise to review the quality and safety aspects of the license application. The joint review option is also under consideration for tuberculosis vaccines [18].

The WHO priority plans for vaccine development until 2012 include dengue vaccines. Written WHO standards exist for live, attenuated dengue vaccines (TRS 932), and several technical consultations have been held to support the science base for dengue vaccine evaluation; these include consultations on dengue vaccine development [19],[20] and guidelines for plaque-reduction neutralization testing of human antibodies to dengue viruses [21]. It is critical that improved standardized tests be introduced as soon as possible for the diagnosis of early infection and for the measurement of immune protection (requiring identification of a correlate of protection). WHO, through its Expert Committee on Biological Standardization, can evaluate and standardize such tests; in addition, WHO, along with its Collaborating Centers, may also help ensure availability of necessary standards and reagents for use in the field. In addition, revised guidelines for the evaluation of dengue vaccines in exposed populations have been published [2].

This paper reports on two meetings that occurred in 2007. In the intervening time (up to November 2010), there has been no consideration of the issues discussed in the paper that would change the conclusions stated in the paper. WHO and PDVI convened a meeting in October 2009 in Bangkok that considered the interactions between scientific regulatory reviews and ethics committee reviews of applications to undertake clinical trials of dengue vaccine. A key conclusion of the meeting was that there needs to be better collaboration between scientific and ethical reviews, but there was no alteration in scientific or technical views about the regulation of dengue vaccines. A manuscript summarizing this meeting is in preparation. Also, since the meetings, two companies (Inviragen and Hawaii Biotech) have launched Phase 1 trials of their vaccines and one company (sanofi pasteur) has launched both Phase 2b and Phase 3 trials. These actions have only served to highlight the conclusions stated in this paper.

## Summary

A number of important issues have been identified for regulatory review of dengue vaccines. Plans are being developed to provide appropriate training and capacity building for developing country NRAs in endemic countries. The issuance in 2008 by WHO of Guidelines for the Evaluation of Dengue Vaccines in Developing Countries is an important milestone in ensuring a sound scientific basis for the clinical evaluation of dengue vaccines. The activities by manufacturers to develop safe and efficacious dengue vaccine candidates together with collaboration among PDVI, WHO, manufacturers, and developing countries to support the planned testing, clinical trial design, and licensure means it is possible that at least one dengue vaccine could be licensed within the next 4–5 years.

## Abbreviations

DCVRN: Developing Countries' Vaccine Regulators Network
DHF: dengue hemorrhagic fever
DSS: dengue shock syndrome
EMA: European Agency for the Evaluation of Medicinal Products
FDA: Food and Drug Administration
IVI: International Vaccine Institute
IVR: Initiative for Vaccine Research
NIH: National Institutes of Health
NRA: national regulatory authority
PDP: product development partnership
PDVI: Pediatric Dengue Vaccine Initiative
WHO: World Health Organization
